# A clinical trial is a success, not a failure, if it does not demonstrate efficacy or does identify safety concerns

**DOI:** 10.1016/j.conctc.2018.10.004

**Published:** 2018-10-22

**Authors:** Simon E. Kolstoe, Hugh Davies, Janet Messer

**Affiliations:** School of Health Sciences and Social Work, University of Portsmouth, Portsmouth, PO1 2FR, UK; Health Research Authority, Ground Floor, Skipton House, 80 London Road, London, SE1 6LH, UK

Dear editor,

As a group involved with the ethics review of clinical trials we enjoyed the article by David Fogel describing the reasons why clinical trials “fail” [1]. We firmly believe that along with protecting participants, an important role of Research Ethics Committees (RECs) is to support researchers in carrying out high quality and successful research [2].

In the UK the Health Research Authority (HRA) coordinates 66 RECs that review around 700 clinical trials each year. Although REC review occurs at the beginning of projects prior to participant recruitment, HRA RECs have developed significant experience in reviewing protocols and identifying issues, especially around experimental design, that may contribute to future “trial failure”. However, from a REC perspective “trial failure” is approximately defined as the failure to complete the research as described, and therefore failure to produce published and relevant scientific or clinical knowledge.

Given this context we disagree strongly with David Fogel who describes the “*primary reason for trial failure*” as “*failing to demonstrate efficacy or safety*”. While we can try to appreciate the perspective of a pharmaceutical company whose aim is to develop a useable and marketable drug, intervention or technology, the entire basis for conducting any form of trial must be a reasonable suspicion of clinical or safety equipoise. Again we do appreciate that regulators insist on certain studies being carried out prior to awarding a marketing authorisation, but even these requested studies are based upon the regulator needing the information on whether or not the proposed intervention (or dosage regime) is at least safe. As a result, although unfortunate from the experimental team's point of view, demonstrating a lack of efficacy, or uncovering previously unappreciated safety issues, represents a form of trial success, not failure.

Our RECs also review trial amendments that are commonly produced as a result of trialists encountering unanticipated problems. We therefore broadly concur with David Fogel on the other factors he lists as leading to significant problems encountered by trialists. We also broadly agree with his observation (under 4. Eligibility Criteria) that: “*Performing a requisite literature review for related studies remains a labour-intensive task requiring personnel with specific knowledge who can interpret the framework, criteria, and results of prior clinical trials.”* BUT we do not think this is a suitable excuse for poor study design and thus trial failure. If trialists are unable or unwilling to complete a thorough literature review (and ideally referencing or conducting a systematic review) prior to commencing a trial, they should not be conducting trials in the first place. Time and again, through our RECs, we see studies with inadequate scientific justifications and poorly designed methodologies caused by incomplete engagement with the literature. Here the answer is not artificial intelligence, it is better scholarship!

## Appendix A Supplementary data

Data Profile Data Profile
